# The forgotten link: how the oral microbiome shapes childhood growth and development

**DOI:** 10.3389/froh.2025.1547099

**Published:** 2025-02-07

**Authors:** Raymond Rubianto Tjandrawinata, Nurlinah Amalia, Yosi Yohanes Putra Tandi, Ariq Fadhil Athallah, Caesaroy Afif Wibowo, Muhammad Reva Aditya, Athaya Rahmanardi Muhammad, Maghfira Rahma Azizah, Farizky Martriano Humardani, Ammar Nojaid, Jeremy Alvaro Christabel, Alfi Agnuristyaningrum, Fahrul Nurkolis

**Affiliations:** ^1^ Center for Pharmaceutical and Nutraceutical Research and Policy, Atma Jaya Catholic University of Indonesia, Jakarta, Indonesia; ^2^ Medical Study Program, Faculty of Medicine, Universitas Brawijaya, Malang, Indonesia; ^3^ Master Program of Biomedical Science, Faculty of Medicine, Universitas Brawijaya, Malang, Indonesia; ^4^ Medical Research Center of Indonesia, Surabaya, Indonesia; ^5^ Bachelor of Medicine, Faculty of Medicine, Universitas Padjadjaran, Bandung, Indonesia; ^6^ Medical Study Program, Faculty of Medicine, Universitas Airlangga, Surabaya, Indonesia; ^7^ Doctoral Program in Medical Science, Faculty of Medicine, Universitas Brawijaya, Malang, Indonesia; ^8^ Master of Basic Medical Science, Faculty of Medicine, Universitas Airlangga, Surabaya, Indonesia

**Keywords:** oral microbiome, stunting, childhood growth, dysbiosis, probiotics, nutrition, malnutrition, gut microbiome

## Abstract

Childhood stunting, defined as impaired linear growth and development, remains a significant global health challenge with long-term consequences on cognitive and physical well-being. Emerging evidence highlights the pivotal role of the oral microbiome—a dynamic microbial ecosystem—in influencing nutritional status, immune response, and overall systemic health. This review explores the intricate interplay between the oral microbiome and stunting, emphasizing mechanisms such as microbial dysbiosis, its impact on nutrient absorption, and immune modulation. Disruptions in the oral microbiome can lead to nutrient malabsorption and systemic inflammation, further exacerbating growth impairments in children. Furthermore, the potential for microbiome-targeted diagnostics and interventions, including probiotics and prebiotics, offers novel strategies to address stunting. A deeper understanding of these interactions may inform innovative diagnostic tools and therapeutic interventions aimed at mitigating stunting through oral microbiome modulation. Integrating oral microbiome research into stunting prevention efforts could provide valuable insights for public health strategies to improve child growth and development, particularly in resource-limited settings. Future research should focus on elucidating the molecular pathways linking the oral microbiome to stunting and developing personalized interventions that optimize microbiome health in early life.

## 1 Introduction

Childhood stunting, defined as a height-for-age measurement below −2 standard deviations of the WHO Child Growth Standards median (1), is a significant public health concern globally. In 2022, there were 148.1 million children globally under the age of 5 who experienced stunting. This issue is especially more common in low-income countries, with an estimated average prevalence of 34.6% (2). In 2022, Indonesia reported a stunting prevalence of 21.6%, with the highest rates observed in children aged 3 to 4 years, which constituted 6%. Some study represents a decrease compared to the stunting rate in Indonesia in 2021, which was 24.4% (3). This condition is associated with increased risk of mortality, morbidity, and suboptimal cognitive and motor development, with long-lasting implications for individual and population health (4).

Stunting caused by various risk factors. Direct factors include food intake, infectious diseases, and child characteristics such as low birth weight (LBW), and food consumption. Indirect factors encompass non-exclusive breastfeeding, access to health services, and family characteristics (parent's occupation, education level, and economic status). The educational level of parents, particularly mothers, can significantly influence the occurrence of stunting by affecting nutritional practices during pregnancy and after childbirth, infection prevention, and parenting styles. Other contributing factors to stunting in children include infections, inadequate sanitation and water supply, household wealth, paternal smoking, maternal age, and parenting practices as household and family factors (5). For children with stunting, interventions may include monitoring toddler growth, providing exclusive breastfeeding for infants under 6 months of age, offering complementary feeding for children aged 6 to 23 months, and ensuring complete basic immunization for infants (6). In the process of addressing stunting, parental involvement is essential, particularly in relation to nutrition provision and infection prevention in children. The absorption of nutrients provided to children can be influenced by several factors, one of which is the presence of the oral microbiome.

The oral cavity is inhabited by a diverse array of microorganisms, commonly referred to as the oral microbiome (7). Research on the oral microbiome has heightened awareness of the critical balance between the host and the microbial species that coexist within it, which is vital for maintaining oral health throughout all stages of life. It is now recognized that changes in the oral microbiota contribute significantly to the development and progression of various oral diseases (8). The oral microbiome is essential for health, as it can lead to both oral and systemic diseases. It exists within biofilms throughout the oral cavity, creating an ecosystem that sustains health in a balanced state. However, imbalances in this equilibrium can allow pathogenic organisms to emerge and cause disease (9). Disruptions to the delicate balance of this microbiome, known as dysbiosis, have been linked to various oral and systemic diseases, including dental caries, periodontitis, and even non-communicable conditions like diabetes and cardiovascular disease (10). Emerging evidence suggests that the oral microbiome may also contribute to the development of stunting in children.

In recent years, there has been growing interest in the potential role of the oral microbiome in the etiology of stunting, particularly in low- and middle-income countries where the burden of this condition is highest (11–13). According to Vonaesch et al. (14), environmental enteric dysfunction (EED) is an inflammatory syndrome believed to contribute to stunted growth in children and is associated with intestinal dysbiosis and nutrient malabsorption (14). Research on the intestinal microbiota and EED indicates that this condition plays a significant role in chronic malnutrition. The study also found that over 80% of stunted children experience small intestinal bacterial overgrowth (SIBO), characterized by an excessive presence of bacteria typically found in the oral cavity. This suggests a transfer of bacteria from the oropharynx to the small intestine. Bacteria isolated from the small intestines of stunted children have been shown to reduce lipid absorption in cell culture models and experiments in mice. This demonstrates a mechanism by which oral bacterial colonization can exacerbate stunting and nutrient malabsorption. Previously, Vonaesch et al. (15) also conducted research it was found that stunting is likely associated with alterations in the microbial community of the small intestine, which is crucial for digestion and nutrient absorption. The majority of stunted children exhibited small intestinal bacterial overgrowth (SIBO) dominated by bacteria typically found in the oropharyngeal cavity and there was a significant overrepresentation of oral bacteria in the fecal samples of these children indicating a transfer of bacteria from the oropharynx to the gastrointestinal tract (15).

This comprehensive review aims to synthesize the current understanding of the relationship between the oral microbiome and childhood stunting, identify potential mechanisms, and discuss diagnostic and intervention strategies. The review's novelty lies in its focus on the understudied link between the oral microbiome and a critical global health issue, stunting, which has significant implications for child development and overall well-being. The findings from this review can inform future research and the development of targeted interventions to address the burden of stunting in vulnerable populations.

## 2 Oral microbiome

### 2.1 Defining oral microbiome

The oral cavity, a vibrant ecosystem teeming with a remarkable community of bacteria, archaea, protozoa, fungi, and viruses, constitutes the oral microbiome (16). This community resides on biotic environments, such as the buccal mucosa, alveolar mucosa, subgingival crevice, the dorsum of the tongue, hard palate, attached gingiva, and tonsils, as well as on abiotic surfaces such as the tooth, dental implants, and dental restorations. Oral colonization niches collectively make up an area larger than a person's palm (17). The oral microbiome is a dynamic and interconnected community that plays a crucial role in maintaining the integrity and health of the oral environment. At the basis of this microbial community lies its delicate balance, which protects the oral cavity against the emergence of harmful pathogens and ensures the proper functioning of the oral cavity (18, 19).

In early life, an infant's microbiota undergoes rapid changes, developing into a stable, adult-like structure with distinct microbial communities. Oral microbiome colonization begins at birth through maternal transmission, diet, and interactions with caregivers, evolving further with primary and permanent tooth development. This microbial establishment interacts with the maturing immune system, shaping postnatal immune functions and physiological development (20). The microbiome continuously evolves over the course of an individual's lifetime in adaptation to changes in the human body, which influence long-term health (21). Interestingly, the significance of the oral microbiome extends beyond the mouth, as growing scientific evidence indicates that this intricate microbial network can exert far-reaching influences on the overall systemic well-being of the host (22), making it a critical area of research with significant clinical relevance. This complex and dynamic community of microorganisms is not only integral to maintaining oral health but is also increasingly recognized for its profound impact on various physiological processes throughout the body. Disruptions to this delicate microbial equilibrium, a condition referred to as dysbiosis, have been implicated in a broad spectrum of health issues, ranging from common oral diseases like dental caries and periodontitis to serious non-communicable systemic conditions, including obesity, diabetes, nonalcoholic fatty liver disease, cardiovascular disease, inflammatory bowel disease, colorectal cancers, rheumatoid arthritis, and Alzheimer disease (18, 19, 22, 23).

It is essential to unravel the complexities of the oral microbiome, comprehend its complex composition, and clarify the intricate interactions that govern its dynamics. By gaining a deeper understanding of the oral microbiome and its intricate relationship with the human body, researchers and clinicians can unlock new avenues for targeted diagnostics and therapeutic interventions, ultimately empowering healthcare professionals to develop more effective strategies for promoting and preserving the overall well-being of individuals and populations (24).

### 2.2 Composition of oral microbiome

The oral microbiome is a remarkably diverse and dynamic ecosystem, composed of a vast array of microbial species that collectively contribute to the maintenance of oral health (25). The microbiome's composition varies in different sections of the digestive tract and is influenced by the distinct environment of each digestive organ. The oral cavity can be categorized into three distinct environments that host similar microbial communities: the gingiva and hard palate, the tongue and throat, and dental plaque (26). Studies on the composition of the oral microbiome indicate that it remains fairly stable over time (27). Bacteria are the most prevalent residents of the oral microbiome, with more than 700 unique species identified so far. These microorganisms flourish in various areas of the mouth, including the teeth, gums, tongue, and saliva, and are vital for sustaining a healthy oral ecosystem. In addition to bacteria, the oral microbiome contains a wide range of other microbes, such as archaea, fungi, viruses, and protozoa. Although these organisms are less numerous than bacteria, they enhance the overall complexity and functionality of the oral microbiome. The intricate interactions and balance among these diverse microbial populations are crucial for preserving the delicate equilibrium of the oral environment (28). Table 1 shows the type of oral microbiome based on its location.

**Table 1:** Type of oral microbiome based on Its location.

Sample location	Major bacterial groups
Buccal mucosa (29)	*Atopobium, Bacilli, Catonella, Pasteurellaceae Prevotellaceae, Streptococcus, Acidobacteriaceae, Xylanibacter, Phocoenobacter, Bacteroidetes, Firmicutes, Proteobacteria, Actinobacteria*
Tongue (30, 31)	*Actinomycetales, Bacilli, Fusobacterium, Lactobacillales, Prevotella, Pasteurellaceae, Peptostreptococcus, Streptococcus, Veillonella, Treponema, Synergistes, Clostridiales, Firmicutes, Proteobacteria, Bacteroidetes, Actinobacteria, Chlorobi, T. denticola, T. forsythia, P. endodontalis,*
Saliva (32)	*N. flavescens, R. mucilaginosa, S. salivarius, Prevotella histicola, Veillonella parvula, Veillonella atypica, S. salivarius, Streptococcus parasanguinis, Actinomycetales, Fusobacterium, Neisseria, Pasteurellaceae, Prevotella, Streptococcus, Tannerella, Veillonella*
Hard palate (33)	*Mogibacterium, Catonella, Gemella, Prevotella, Streptococcus*
Supergingival and subgingival plaques (34)	*Betaproteobacteria, Corynebacterium, Capnocytophaga, Corynebacterium, Firmicutes, Fusobacterium, Neisseriaceae, Pasteurellacea, Prevotella, Streptococcus, Granulicatella, Porphyromonas, Actinomyces, Neisseria, Treponema, Denticola, Tannerella forsythia, E. faecium*
Throat (20)	*Actinomyces, Firmicutes, Fusobacterium, Pasteurellaceae, Streptococcus*
Tonsils (26)	*Firmicutes, Fusobacterium, Mogibacterium, Pasteurellaceae, Prevotella, Streptococcus*

**Figure 1:**
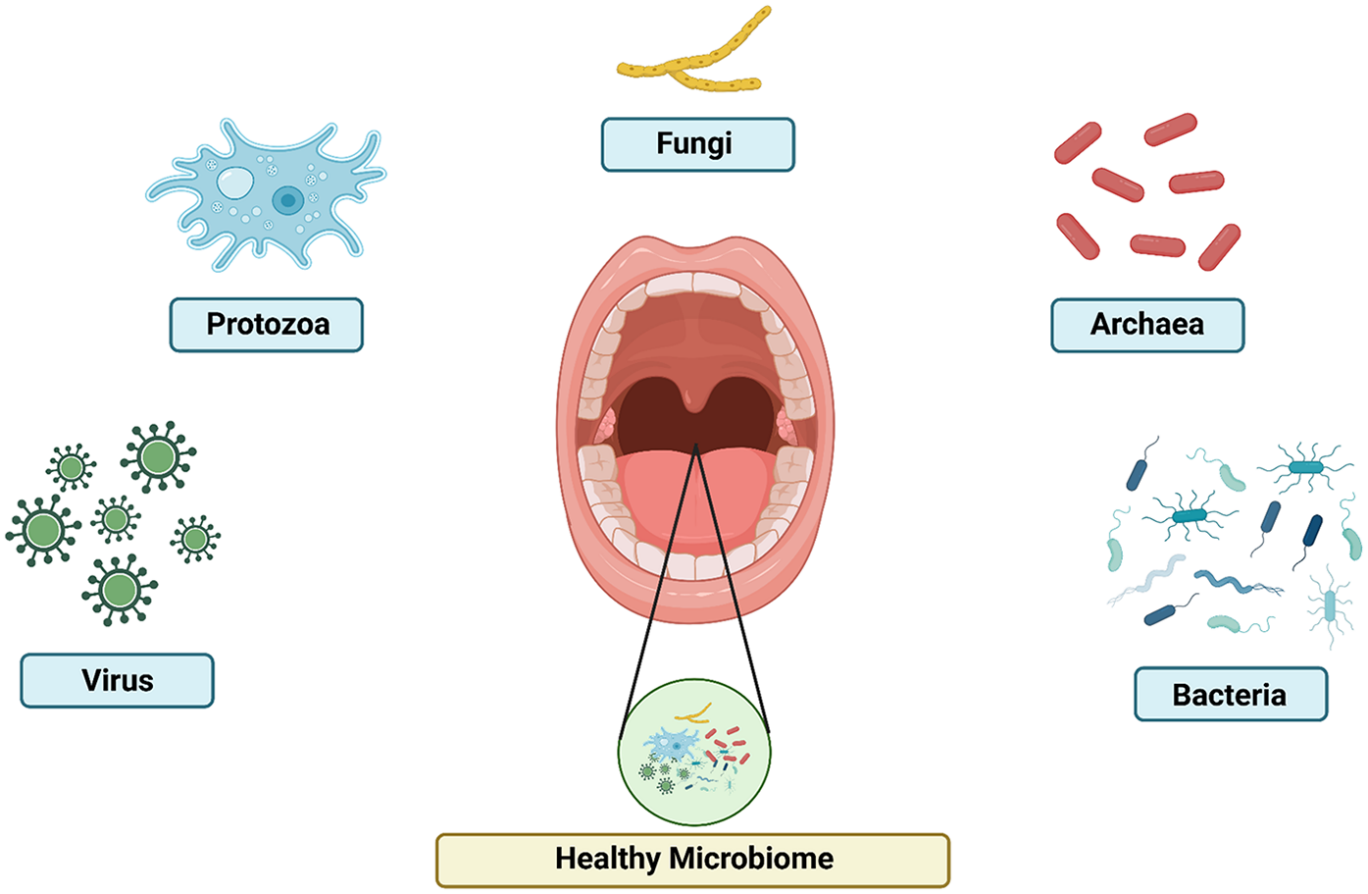
The composition of oral microbiome. Healthy microbiome include virus, protozoa, fungi, archaea, and bacteria with the optimum level of each other.

This microbial community is inhabited by a range of microorganisms, including bacteria, archaea, fungi, viruses, and protozoa, each with their unique roles and interactions within the oral cavity, as shown in Figure 1 (18–20). The Human Oral Microbiome Database (HOMD) is a curated 16S rRNA database that includes taxa currently recognized and contains sequences of oral bacterial genomes (35). Most of these are considered oral commensals. The oral microbiome also contains a small number of archaebacteria, primarily methanogens (36). These approaches have made it possible to establish a comprehensive database covering all known oral bacterial species. The fungal microbiota can be analyzed through high-throughput sequencing of Internal Transcribed Spacer 1 (ITS1) amplicon libraries. The fungal mycobiome consists of species such as *Candida, Cladosporium, Alternaria, Aspergillus, Eurotium, Fusarium, Cryptococcus, Filobasidiella, Aureobasidium,* and *Malassezia* (37). The protozoan population is thought to be primarily saprophytic, with the most frequently identified species being *Entamoeba gingivalis* and *Trichomonas tenax*. *E. gingivalis* (38). The viruses most frequently found in the oral cavity include rotavirus, norovirus, HIV, hepatitis C virus, herpes simplex viruses 1 (HSV1) and 2 (HSV2), Epstein–Barr virus, and influenza viruses. In addition to these commonly identified viruses, there are also some less prevalent viruses, such as eukaryotic DNA viruses (20).

### 2.3 Factors influencing oral microbiome

**Figure 2:**
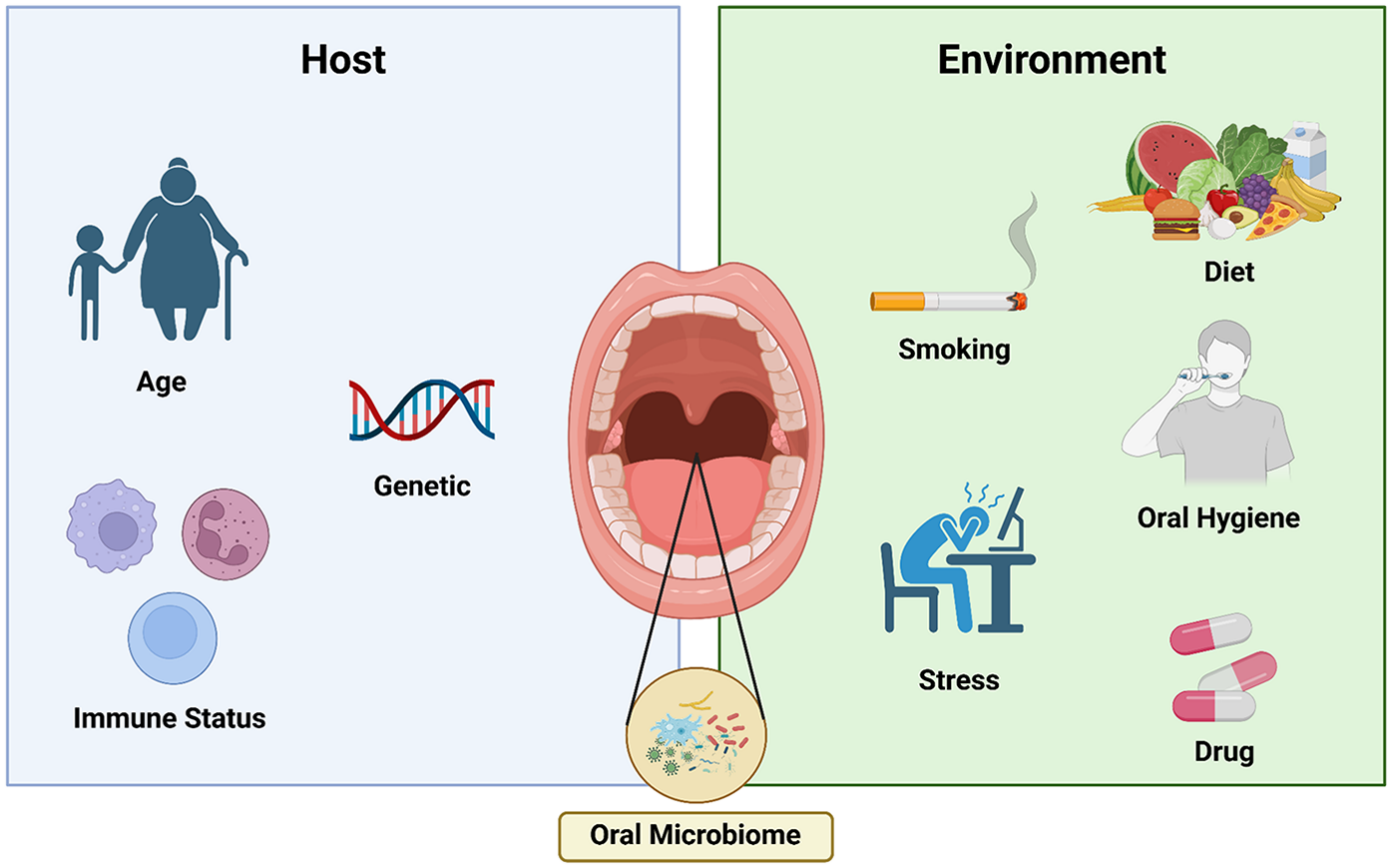
Factor influencing oral microbiome. Both of host related and environment.

The composition and dynamics of the oral microbiome are influenced by a multitude of factors, both host-related and environmental as shown in Figure 2. Host factors, such as age, genetics, and immune status can shape the development and evolution of the oral microbiome over the course of an individual's lifetime (39). The host's age was found to have a two-way relationship with oral microbiomes. Older individuals were found to have reduced diversity in microbiota and shifted species dominance from the beneficial microbiota species to harmful ones. These shifts in oral microbiome could in turn induce degenerative and chronic diseases that potentially cause negative effects towards aging (40). Host immunity was discovered to play a crucial role in the aforementioned microbiota diversity by defending the oral microbiome from pathological species so that beneficial species can flourish. Genetics could potentially have a considerable impact on the oral microbiome as it affects the variability of other intrinsic factors that could affect the microbiome, such as an individual's saliva composition, the physical property of oral hard tissues, the host's immune response, and the composition of microbiomes in different anatomical oral locations (41). Various oral diseases, such as dental caries and periodontitis, have also been found to affect dysbiosis in the oral microbiome as it promote the growth of pathological microbiota species (42).

Environmental factors, including diet, oral hygiene practices, and exposure to various chemicals and medications, can also profoundly impact the delicate balance of the oral microbial community (19, 39). The relationship between microbiome and host is dynamic, and influenced by many aspects of modern lifestyle, such as diet, tobacco consumption and stress, which can alter our microbiome and its properties, and induce a state in which this finely tuned ecosystem is no longer in balance (18, 19). Physical and psychological stress plays a role in decreasing the richness and diversity of the oral microbiome as it was evident in research conducted on two population that have significantly different stress levels. The research showed that the population with lower stress levels has more diverse and rich oral microbiome compared to the population with higher stress levels (43). Different human lifestyles have also been found to vary the oral microbiome according to research comparing the oral microbiome in American and European populations which mostly have modern lifestyles with the oral microbiome in Nepalian populations which have more traditional lifestyles. The research showed that there was a significant difference in oral microbiome between populations with modern lifestyles in America and Europe compared to traditional lifestyles in Nepal with populations with modern lifestyles having less diversity than populations with traditional lifestyles. The research also compared the oral microbiome between various populations in Nepal and the comparison result showed no significant changes in the oral microbiome diversity (44).

To address this divergence and maintain a harmonious state to protect health and prevent disease, we must not focus on the host and its residents as separate units, but instead consider the holobiont as one (18, 19).

### 2.4 Oral microbiome and child development

**Figure 3:**
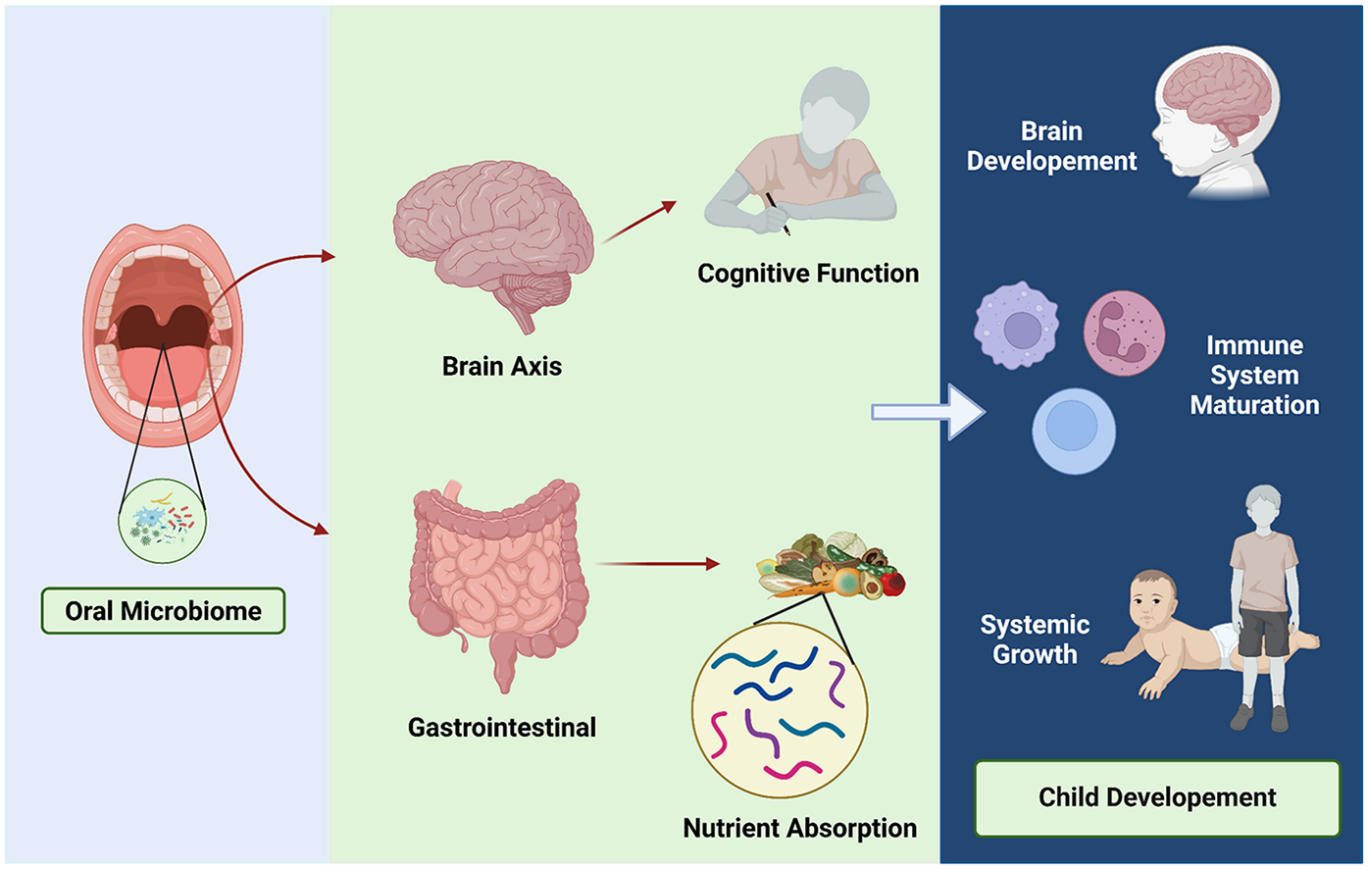
Oral microbiome and child development. Healthy microbiome involving on child development through brain axis and gastrointestinal contribution.

The oral microbiome plays a critical role in both local and systemic health, with increasing evidence highlighting its significance in child development and growth (Figure 3). Among the key areas of interest is the potential relationship between the oral microbiome and childhood stunting, a global health challenge characterized by impaired linear growth and delayed development. Stunted children often exhibit alterations in their microbial composition, pointing to a possible role of microbial imbalances in this condition (18, 19, 39).

One notable phenomenon observed in stunted children is microbial decompartmentalization, where oral bacteria abnormally colonize the small intestine and fecal microbiota, disrupting the natural compartmentalization of the gastrointestinal microbiome. This shift is associated with a significant reduction in beneficial bacteria, such as butyrate producers, which are essential for maintaining intestinal barrier integrity, nutrient absorption, and overall gut health. The ectopic presence of oral bacteria in the small intestine has been linked to lipid malabsorption and increased intestinal permeability, both of which aggravate nutritional deficiencies and impair systemic growth. Experimental studies further demonstrate that this abnormal colonization triggers profound metabolic disturbances, suggesting a direct role in the pathophysiology of stunting (14, 15).

Beyond its role in linear growth, the oral microbiome is intricately connected to broader aspects of child development, including cognitive function, immune system maturation, and neurological health. The oral microbiome-brain axis, for example, suggests that oral bacteria produce neuroactive compounds and modulate immune responses, potentially influencing cognitive development (45). Disruptions in this axis may contribute to delayed cognitive and neurological outcomes in stunted children (39, 46). Similarly, the oral microbiome plays a pivotal role in shaping immune tolerance and defense mechanisms, with dysbiosis increasing the risk of systemic inflammation and infections that can indirectly impair growth and development (19). Early interactions between the microbiome and the host during neonatal development are crucial for establishing innate and acquired immune functions, as well as physiological development, all of which influence long-term health.

Understanding the intricate interplay between the oral microbiome and child development opens new avenues for preventive and therapeutic strategies. Interventions aimed at promoting a healthy oral microbiome, such as improved oral hygiene, probiotic therapies, and adequate nutrition, could potentially mitigate the risk of stunting and enhance child health outcomes. Future research should focus on elucidating the molecular pathways linking the oral microbiome to stunting and identifying effective, context-specific interventions to address this global health challenge (19, 39, 46).

## 3 Stunting

### 3.1 Stunting in children

Childhood stunting, defined as a height-for-age measurement below two standard deviations of the WHO growth standards, is a significant global health challenge. Affecting an estimated 144 million children worldwide, stunting is a multifactorial condition that can have lasting consequences on an individual's physical and cognitive development. During the first 1,000 days of life, also known as the golden period, the human brain undergoes rapid and advanced growth, requiring adequate and complex nutrition to support the neurogenesis, leading impaired cognitive skills. Essential macronutrients include protein, omega-3 fatty acids, carbohydrates, vitamins, minerals, and water for neurodevelopment during the golden period. While certain micronutrients, such as iron, zinc folic acid, and B12 also have shown its critical role to support the development (47).

Although the known primary suspect causes of stunting are nutrition-related, its underlying etiologies are complex and multifaceted, involving a combination of factors such as inadequate nutrition, poor sanitation and hygiene, recurrent infections, and environmental enteropathy. For instance, inadequate nutrition stems from other predisposing factors, ranging from food insecurity and lack of dietary diversity, to insufficient breastfeeding practices. On the other hand, access to clean water and poor hygiene & sanitation shows a strong relationship with the development of stunting (48). This particular factor leads to long-term exposure to environmental pathogens, which causes chronic infection and gut dysbiosis (15). For instance, open defecation and contaminated water expose children to various pathogens, further exacerbating nutritional deficiencies. Additionally, chronic and recurrent infections, especially affecting gastrointestinal tract, are closely related to stunting. Frequent diarrhea leads to impairment of nutrient absorption which causes malnutrition and illness. Another critical factor is environmental enteropathy, a condition of intestinal inflammation without overt symptoms. While the children tend to be asymptomatic, there's an ongoing nutrient malabsorption due to inflammation and microbiome dysbiosis of the gut (15, 49).

Emerging evidence suggests that the oral microbiome may be a key player in the etiology of stunting (19, 39). The oral microbiome, with its influence on nutrient absorption, immune function, and the production of metabolites, may contribute to the impaired linear growth observed in stunted children. Oral microbiome is highly associated with nutrient digestion and absorption. For instance, certain bacteria within the oral cavity, such as *Lactobacillus spp.* can enhance bioavailability of essential nutrients by breaking down complex food components, therefore increasing the absorption in the gastrointestinal tract (50). Moreover, specific microbial species—are known to aid in the fermentation of dietary fibers and carbohydrates, producing short-chain fatty acids (SCFAs) which promote gut health and improve nutrient uptakes (51). Hence, the disruption of microbiome plays a role in the development of stunting. Studies have highlighted microbiome association with malnutrition, while chronic undernutrition remodel microbiome composition—which leads to dysbiosis, changes of the microbiome disrupt many physiological function which further worsened stunting. Despite optimal nutritional interventions, the normalization of linear growth in children living in resource-constrained settings has remained an elusive goal. This observation has led researchers to explore alternative factors, such as the role of the oral microbiome, in the complex pathogenesis of stunting (52).

### 3.2 Malnutrition and stunting

Malnutrition, which encompasses both undernutrition and overnutrition, significantly contributes to childhood stunting. Inadequate intake of essential macro- and micronutrients can lead to impaired growth and development, with long-lasting consequences on an individual's physical and cognitive abilities. Humans have the capability to adapt under several circumstances of malnutrition state, such as gene expression comprising the physiological and nervous system (neurogenesis, neural plasticity, blood pressure, behaviour), immune cell (immunodeficiency or blunt immune response) (53), metabolism response (energy expenditure, energy storage, enzyme physiology), and more to cell equilibrium (electrolyte imbalance, apoptosis, atrophy, cell coding protocols) (54). However, the prolonged detrimental condition or unsustained incapability to overcome the condition, especially in the children population, alters the physiology of the body by leading to a detrimental state that is susceptible to hypoglycemia, hypothermia, dehydration, and infection (55).

Several timelines of life contribute significantly to children's growth and development, beginning with preconception, prenatal gestational state, and post-partum (neonates, infant). The preconception stage from paternal and maternal perspectives promotes various readiness and linkages to heritability. Several studies have stated that pathological conditions such as malnutrition (underweight or overweight-obesity) evaluated through body mass index (BMI), diet/nutrient, and associated weight gain provide a large exposure to anatomical, cognitive, and mental anomalies during the prenatal stage and even until post-partum (56). Interventions that are considered to have a significant impact during the pregnancy interval are those that occur as early as possible during fetal life, thereby reducing the risk of stunting at the age of up to 4.5 years. Therefore, maternal malnutrition, particularly during pregnancy and lactation, can initiate the process of linear growth faltering even before birth, leading to intrauterine growth restriction and low birth weight (4).

Suboptimal feeding practices in infancy, such as a lack of exclusive breastfeeding or inadequate complementary feeding, can further exacerbate the risk of stunting. Evaluation in low-income populations shows that exclusive breastfeeding gives proper protection against stunting risk. The assessment of stunting risk is associated with age, maternal occupation, monthly expenditure, and nutrition. In early life, especially in low-income populations, the economic requirement increases significantly, assuming that exclusive breastfeeding contributes to a decrease in monthly money. Exclusive care by biological mothers also reduces the risk of stunting by 2.5 times, with the additional consideration that biological mothers found to be 4 times more intense and attentive in providing breastfeeding (4). The critical stage after the gestational stage is known as the first 1,000 days of life of the post-partum stage. The statement was also proven by the main source of nutrition within 6 months being closely related to lactation, such as macro- and micro-nutrients, even contained with immunoglobulin (IgA, IgM, IgG) concentrated in colostrum and mature milk. Structurally, immunoglobulins and various protein chains are susceptible to environmental exposure, labile, or require invasive routes to prevent protein structural degradation (57).

Suboptimal nutrition during neonate and infant is linked to global malnutrition or even early supplementary food, along with an impact on gastrointestinal anatomy-physiology directly. In addition to nutritional factors, a high burden of infectious diseases, particularly in resource-constrained settings, has been strongly linked to the development of stunting. Macro- and micro-nutrient deficiencies in stunted children are implicated with inadequate nutrition and psychosocial stimulation, may lead to incapability with cognitive function or even infection susceptibility. Those nutrients not only supply the demand of human body metabolism but also support the immune system. Acute or chronic deficiency affects not only the growth but also the immune system, implying the reason for onset, frequency, and duration regarding the condition of malnutrition that further worsens the systemic condition (58). However, the infection affects bidirectional stunting in children. Immunocompromised and or more pathogens exposure in the children population raises the risk of being infected. Infection increases the metabolic source requirement and impairs the physiology, causing many interfactorial problems and contributing to stunting conditions (59).

### 3.3 Oral health and stunting

Recent research has highlighted the potential role of the oral microbiome in the development of childhood stunting. A study by Judijanto et al. (60) has done stool sample analysis indicating that children experiencing stunted growth had lower gut microbiota diversity compared to children with normal growth patterns. Furthermore, longitudinal tracking of growth outcomes showed that higher initial gut microbiota diversity was associated with more positive growth trajectories. Notably, the study identified gut microbiota diversity as an independent predictor of stunting, even when socioeconomic status, diet, and environmental factors were taken into account (60).

Infants who are not exclusively breastfed are more susceptible to diarrhea and asymptomatic enteric infections, which may be attributed in part to the transmission of infectious agents during bottle-feeding (12). This finding is supported by a study in Dili which revealed that bottle feeding was associated with significantly higher odds of pathogen detection compared to exclusive breastfeeding at home. The most frequently detected pathogens were diarrheagenic *Escherichia coli* and *Campylobacter spp*. (61). Several research suggest exclusive breastfeeding as a protective alternative towards diarrhea. Early initiation of breastfeeding, exclusive breastfeeding, and avoiding bottle feeding are associated with lower rates of acute respiratory infection and diarrhoea in Ethiopian infants and young children. Furthermore, longer durations of exclusive breastfeeding offer greater protection against a wide range of infectious diseases (49, 62).

Additionally, poor oral hygiene and the subsequent colonization of the oral cavity by harmful bacteria can lead to the development of dental caries and periodontal diseases, which have been associated with impaired linear growth in children. Early Childhood Caries (ECC) is associated with less favorable ponderal growth, as evidenced by lower weight-for-height Z-scores over time. Additionally, oral health-related quality of life, particularly eating difficulties, mediates the negative relationship between dental caries and child growth in Bangladeshi children aged five to nine years. Dental caries in primary dentition has also been linked to undernutrition, potentially hindering healthy weight gain. Furthermore, undernutrition itself is associated with increased odds of developing early childhood dental caries in preschool-aged children (63).

The oral microbiome, through its influence on nutrient absorption, immune function, and the production of microbial-derived metabolites, may contribute to the complex pathogenesis of stunting. Undernutrition appears to have intergenerational origins, with the microbiome playing a potential mediating role. Transplacental signals during pregnancy may influence fetal development, while vertical transmission of the microbiome during birth, breastfeeding, and regular skin contact in early life further shape the infant's microbiome. A healthy developmental trajectory of the gut microbiome is essential for supporting optimal child growth and development. However, disruptions such as food insecurity, unhealthy diets, poor hygiene, and inadequate sanitation can negatively impact maternal and child health, contributing to undernutrition phenotypes such as stunting, wasting, and micronutrient deficiencies. Many of these adverse effects may be linked to an impaired gut microbiome (64).

**Figure 4:**
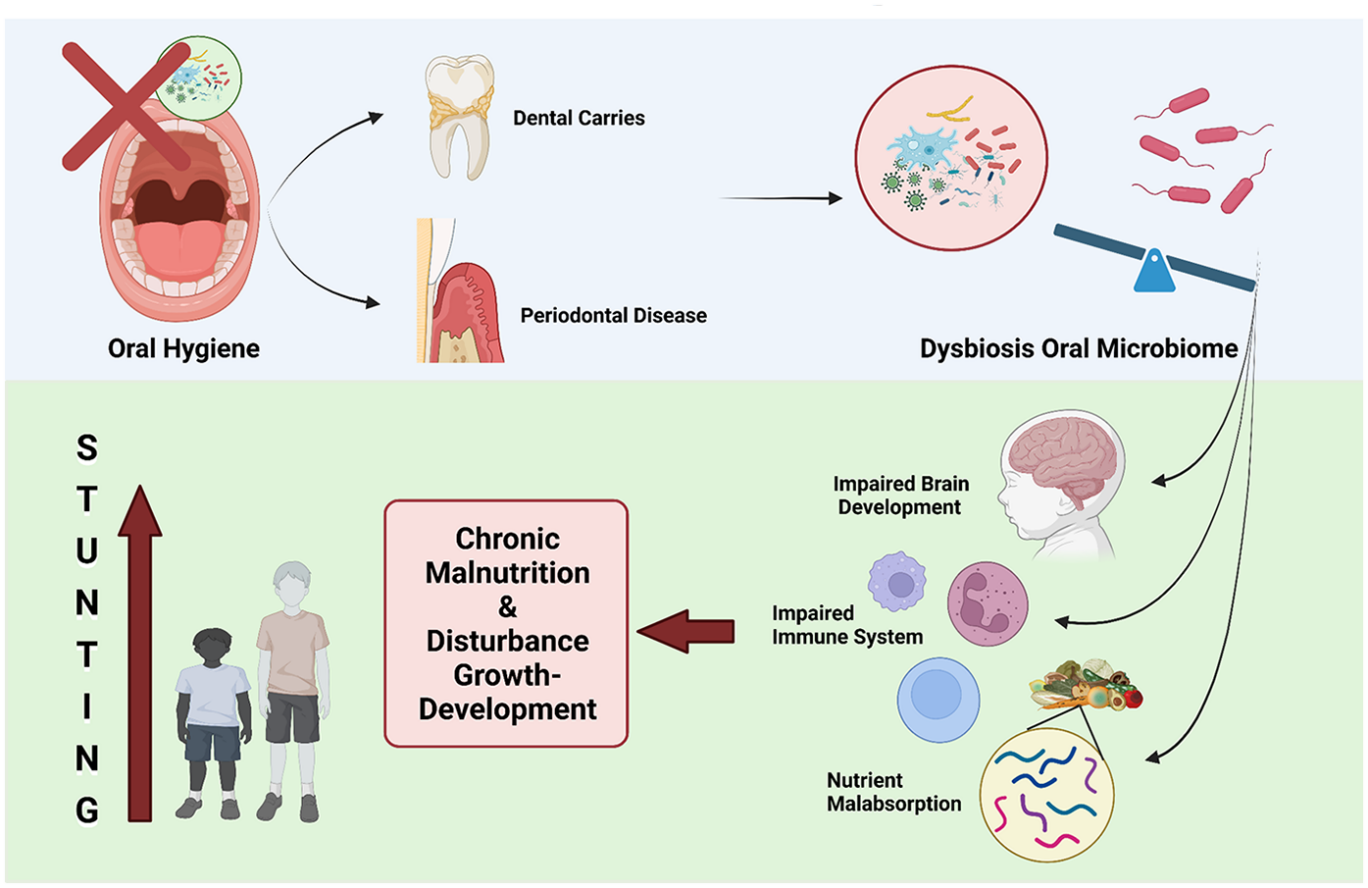
Oral health and stunting. Dysbiosis of oral microbiome leading to stunting because of chronic malnutrition.

Further research is needed to elucidate the precise mechanisms by which the oral microbiome interacts with other factors, such as nutrition and infection, to impact childhood linear growth and development as shown in Figure 4. Specifically, longitudinal studies investigating how changes in the composition and function of the oral microbiome correlate with nutritional intake, systemic infections, and immune responses could provide valuable insights. Research exploring the bidirectional relationship between malnutrition and the dysbiosis of the oral microbiome may uncover how deficiencies or imbalances in key nutrients alter microbial communities, potentially affecting growth outcomes. Additionally, studies employing multi-omics approaches, such as metagenomics, metabolomics, and proteomics, would help clarify the pathways through which microbial metabolites influence systemic processes related to growth and development. Given the complex interplay between environmental, genetic, and microbial factors, interdisciplinary studies involving pediatricians, microbiologists, nutritionists, and public health experts are essential. This integrative approach could lead to targeted interventions, such as probiotics or dietary modifications, to support optimal growth trajectories in children, particularly in populations vulnerable to growth faltering. In summary, the oral microbiome has emerged as a promising area of investigation in the quest to understand and address the global burden of childhood stunting (64).

### 3.4 Gut microbiome and stunting

While the oral microbiome has been the focus of recent research on stunting, the gut microbiome also plays a crucial role in child growth and development. The gut microbiome, which is established early in life, can be significantly impacted by factors such as mode of delivery, antibiotic exposure, and diet. Dysbiosis in the gut microbiome has been linked to various health conditions, including malnutrition and stunting. Well, as we know the gut microbiome represents a sophisticated ecology that helps its host perform vital tasks. This definition signifies a significant enhancement of the concept of a microbial community, as it delineates a microbial community characterized by unique traits and functions, together with its interactions with the environment, leading to the establishment of specialized ecological niches. The close relationships between hosts and their associated microorganisms gave rise to the theory of coevolution of the host and its associated microbiota (65).

The human gastrointestinal system contains microbial communities comprising 10^3^–10^4^ cells. The colon displays the largest concentrations, as the conditions of pH 5.5–7, extended transit duration, and ample nutrient availability substantially enhance bacterial growth. The colonic bacteria are primarily anaerobic and engage in many metabolic processes, some of which benefit the host. Gut microbiota ferment non-digestible carbohydrates, generating short-chain fatty acids (SCFAs) for the host. Short-chain fatty acids (SCFAs) are known to provide numerous health benefits to the host, including energy provision to epithelial cells, lowering the pH of the intestinal lumen to hinder the growth of specific pathogens, and exerting an anti-inflammatory effect on the host. The gut microbiota is acknowledged for its crucial function in the absorption, storage, and utilization of dietary energy, along with the creation of vitamins K and B12 (66).

This interplay between the oral and gut microbiomes highlights the need for a comprehensive understanding of the role of the human microbiome in childhood stunting. Emerging evidence suggests that the gut microbiome can influence nutrient absorption, immune function, and the production of metabolites that may impact linear growth. Furthermore, the gut microbiome may interact with the oral microbiome, as the two are connected through the gastrointestinal tract. Correlation between gut microbiome and stunting are complex. Emerging data indicates that a subclinical condition of the small intestine is marked by diminished intestinal villi, heightened gut permeability, and microbial translocation into the bloodstream, leading to both localized and chronic inflammation as well as nutrient loss (67). Stunting is a manifestation of chronic malnutrition, which can result from inadequate intake of essential nutrients for growth and development or from chronic illnesses. This inadequate intake may stem from gastrointestinal issues in children, such as impaired villi function affecting nutrient absorption and imbalances in the gut microbiota. Gut microbiota dysbiosis can lead to the loss of critical nutrients. Additionally, chronic illnesses, such as gastrointestinal tract inflammation, are strongly associated with gut microbiota dysbiosis. These conditions divert nutrients that should support growth and development toward addressing the chronic inflammation instead (68).

**Figure 5:**
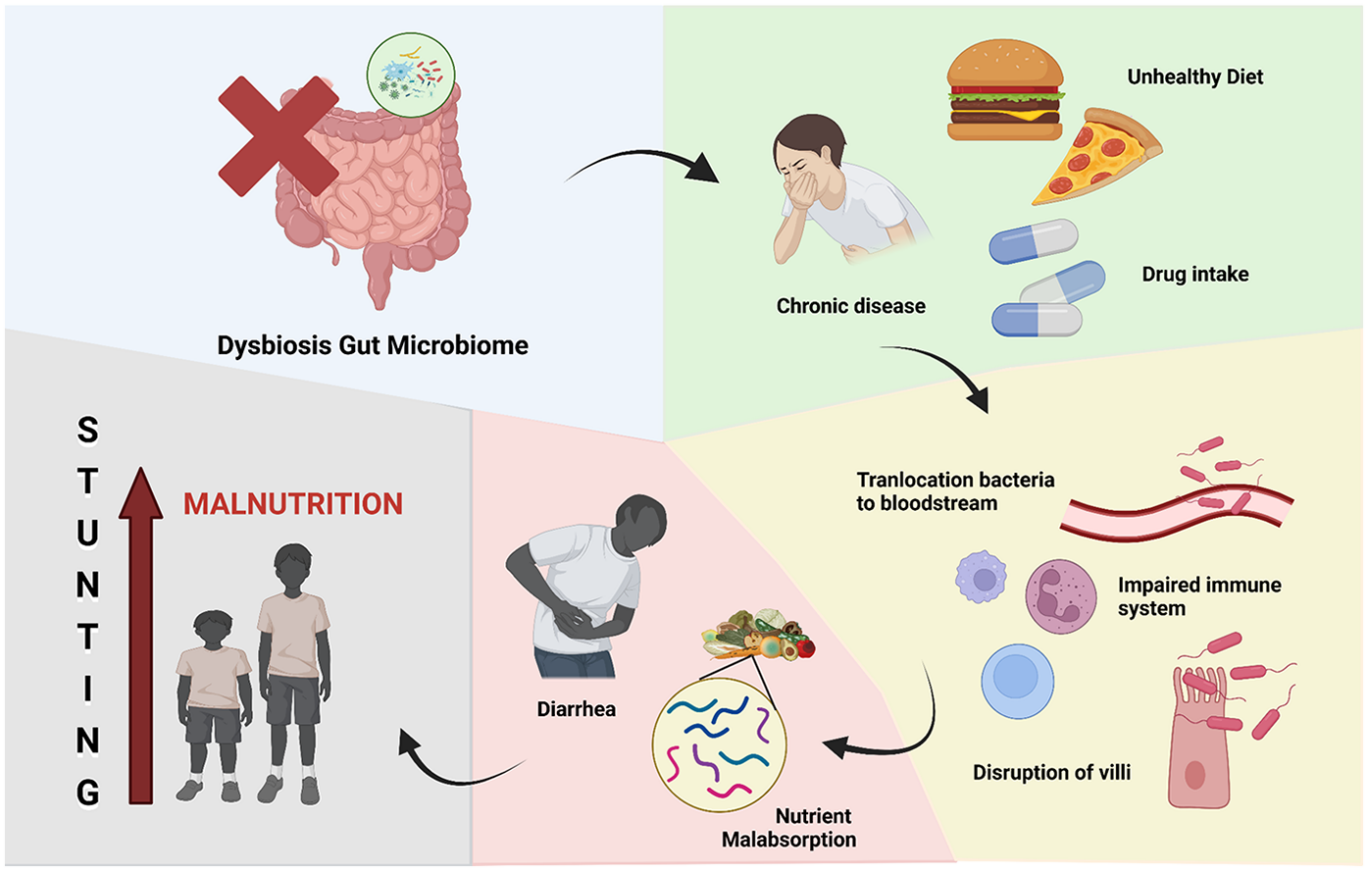
Gut microbiome and stunting. Dysbiosis of gut microbiome leading to stunting because of chronic malnutrition.

Malnutrition and diarrhea were attributed to alterations in the gut microbiota until a few decades ago, as shown in Figure 5. Diarrheal sickness can result in malnutrition due to diminished nutritional absorption and mucosal injury, in addition to nutrient loss linked to each diarrheal episode. Furthermore, findings from the Global Enterics Multicenter Study (GEMS) conducted in West and East Africa, as well as Southeast Asia, indicated that moderate-to-severe diarrhea in children results in reduced bacterial diversity and modified microbiota composition. The initial phase of gut microbiota dysbiosis in malnourished children is believed to involve the reduction of *Bifidobacteria*, subsequently leading to the colonization by potential microbial pathogens such as *Streptococcus sp.*, *Fusobacterium mortiferum*, and *Escherichia coli*, resulting in diarrhea and malabsorption of vital nutrients. Furthermore, it has been demonstrated that malnutrition in children correlates with a decrease in the species *Bifidobacterium longum* and *Lactobacillus mucosae* (69).

In conclusion, the potential link between the oral microbiome and childhood stunting is a promising area of research that warrants further investigation. By elucidating the complex mechanisms by which the oral microbiome contributes to linear growth impairment, new strategies for prevention and intervention may be developed to address this global health challenge (70). The development of microbiota-centered therapeutics has been fueled by the realization that modifications in the composition of the microbiome might impact the effectiveness of treatments and chronic human diseases (71).

## 4 Interplay of oral and Gut microbiome

**Figure 6:**
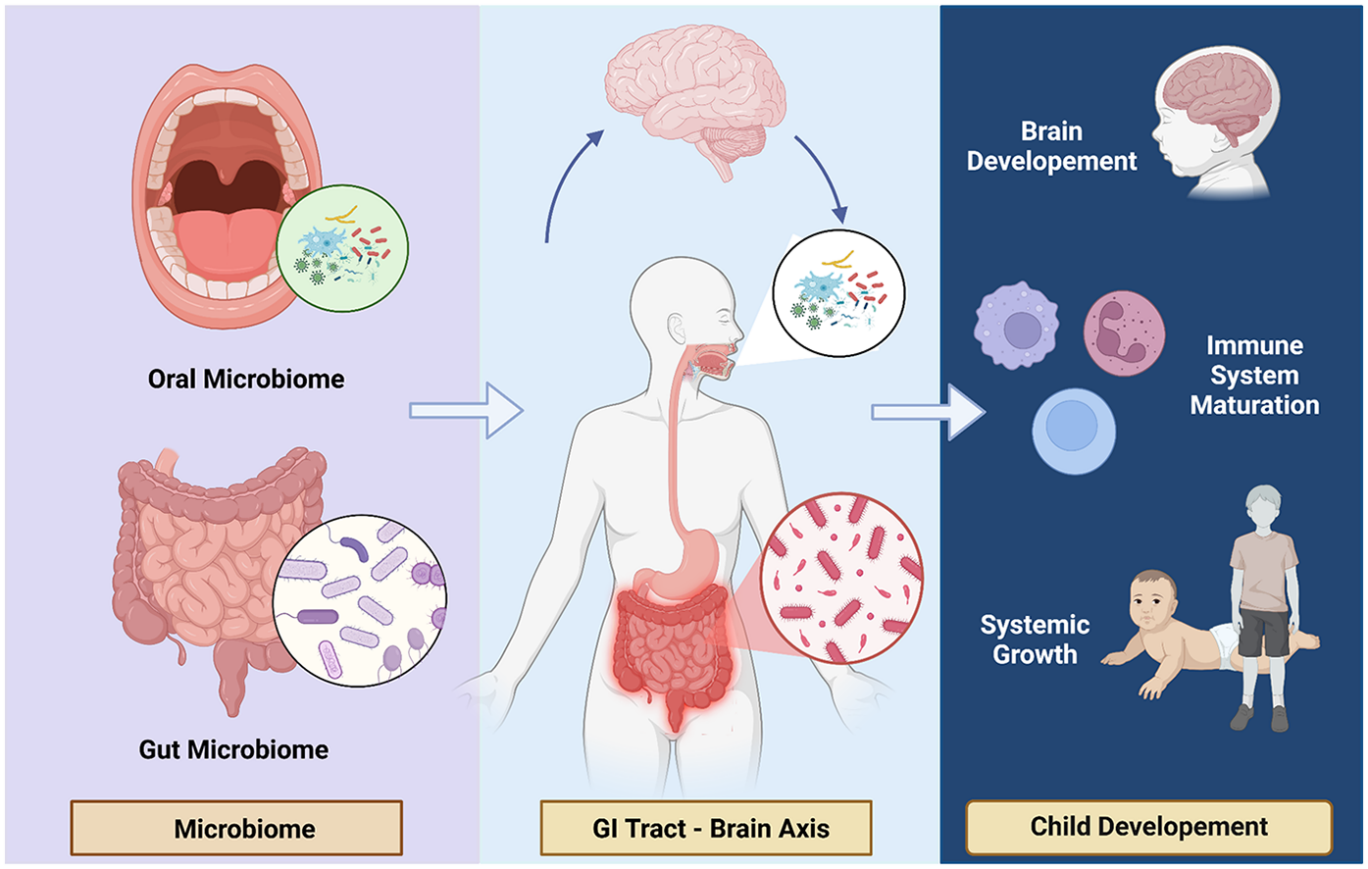
Interplay of oral & Gut microbiome. Oral and gut microbiome closely intertwined to the gastrointestinal tract and brain axis.

The oral and gut microbiomes are closely intertwined, with the oral cavity serving as a gateway to the gastrointestinal tract (Figure 6). The composition and maturity of the gut microbiome are influenced by the microbial communities residing in the oral cavity. Oral microbiota can influence gut microbiota through three primary routes: enteral, hematogenous, and immune cell migration. The enteral route involves the daily swallowing of 0.75–1.5 L of saliva, which contains numerous oral bacteria. Disruptions in the oral microbiome, such as the overgrowth of pathogenic bacteria or the loss of beneficial species, can have downstream effects on the gut microbiome (72). Typically, the oral and gut microbiomes are distinct due to physical and chemical barriers, such as gastric acid and bile. However, certain conditions, including barrier dysfunction, can facilitate microbial translocation. For instance, in neonates and the elderly who often have immature or compromised barriers, they exhibit evidence of oral bacteria in the gut. In some pathological conditions like inflammatory bowel disease (IBD) or colon cancer, oral microbes can also colonize the gut. The hematogenous route, on the other hand, enables oral bacteria to enter systemic circulation through mechanical injuries caused by daily dental activities (e.g., brushing, mastication) or dental procedures. In cases of periodontitis, vascularization and gingival ulceration allow bacteria, such as *Fusobacterium*, to enter the bloodstream, potentially reaching gut tissues or colon tumors. The immune cell migration route offers another mechanism, where oral bacteria survive intracellularly in dendritic cells or macrophages and use them as carriers to the gut. Furthermore, even under healthy conditions, oral microbes like *Streptococcus* and *Prevotella* have been detected in fecal samples, emphasizing that oral-to-gut translocation occurs more extensively than previously anticipated (73).

Conversely, changes in the gut microbiome, driven by factors such as diet, antibiotic use, or enteric infections, can also impact the oral microbiome. This is commonly seen in developing regions due to poor hygienic status and among immunocompromised individuals. Research also demonstrated that variations in gut microbiota profiles, influenced by a long-term fat-enriched diet, correlated with changes in oral microbiota in mice. This bidirectional relationship between the oral and gut microbiomes suggests that a comprehensive understanding of the human microbiome is necessary to elucidate its role in the development of childhood stunting. Recent studies have identified specific bacterial taxa, such as Faecalibacterium prausnitzii, that exhibit consistent covariation with age and are predictive of ponderal growth throughout the first two years of life. These findings suggest that the maturation of the gut microbiome, driven by the interplay between the oral and gut microbial communities, may be a key factor in influencing linear growth and development (72).

In addition to the direct impact of the oral and gut microbiomes on nutrient absorption and immune function, the production of microbial-derived metabolites has emerged as a potential mechanism by which the microbiome can influence childhood stunting. These metabolites, such as short-chain fatty acids and vitamins, can have far-reaching effects on host physiology, including the regulation of growth and development. In summary, the interplay between the oral and gut microbiomes is a critical component in understanding the complex pathogenesis of childhood stunting (74).

## 5 Role of oral microbiome in malnutrition in children

The oral microbiome is distinct from the gut microbiome (75). Interestingly, there are differences between the oral microbiomes of children and adults. Adult oral microbiomes are predominantly influenced by oral health habits, while those of children are shaped by biological sex and weight status (57). Current evidence suggests a link between dental caries and periodontal disease and stunting (76). The colonization of the oral cavity by pathogenic bacteria can lead to the development of dental caries and periodontal diseases. In dental caries, acidic attacks from cariogenic bacteria in the dental plaque biofilm on the tooth surface are triggered by low pH levels, enabling the bacteria to metabolize fermentable carbohydrates. The formation of cariogenic biofilm is a result of microbiome dysbiosis, where the acidic environment (low pH) promotes the growth of certain bacterial species while suppressing acid-sensitive bacteria. This oral microbiome dysbiosis is also observed in periodontal disease, suggesting that the oral microbiome may play a role as a potential contributor to childhood stunting (77).

Essentially, ingested microbes can initiate two overlapping and interacting pathways that contribute to linear growth impairment. Clinical studies have identified ectopic colonization of the small intestine by oral bacteria, which may lead to a reduction in butyrate-producing bacteria in the gut. This reduction in butyrate producers has been associated with stunting, as demonstrated in clinical research. Firstly, partial villous atrophy caused by the presence of these microbes reduces the absorptive surface area and results in the loss of digestive enzymes, leading to maldigestion and malabsorption of essential nutrients. This mechanism is driven by a lack of butyrate, which triggers inflammation and apoptosis, ultimately causing structural changes in the intestine (78).

Secondly, microbes and their products can increase gut permeability by disrupting the intestinal cell layer, allowing luminal contents to translocate into the systemic circulation. This leads to chronic immune activation due to reduced levels of butyrate-producing bacteria, which are responsible for anti-inflammatory responses that help resolve inflammation and maintain gut homeostasis. As a result, nutrient resources are diverted away from growth and development, causing further damage to the intestinal mucosa and exacerbating the issue (79). Moreover, the oral microbiome can influence the gut microbiome through the gastrointestinal tract, a phenomenon known as the oral-cavity-driven gut microbiome, as the two are closely interconnected. Disruptions in the oral microbiome may have downstream effects on the gut microbiome, potentially leading to further impairment of nutrient absorption and immune function, both of which are key determinants of linear growth in children (80).

The Table 2 presents a comprehensive overview of clinical studies exploring the relationship between the oral microbiome and malnutrition in children. These studies examine various populations, including children with protein-calorie malnutrition, hospitalized malnourished children, and those receiving oral nutritional supplements (ONS). Findings from Osatogbe et al. (81) indicate an increase in *Streptococcus* and *Enterococcus* levels in malnourished children, suggesting a link between malnutrition, bacterial infections, and increased susceptibility to diseases. Similarly, Osatogbe et al. (82) reported that hospitalized malnourished children exhibited an overgrowth of pathogenic bacteria such as *Haemophilus influenzae*, *Staphylococcus aureus*, and *Escherichia coli*, leading to conditions like periodontal disease, dental caries, and systemic infections. Fathallh et al. (83) highlighted the role of dietary habits, showing that high carbohydrate consumption promotes the growth of *Streptococcus mutans* and *Streptococcus sobrinus*, contributing to dental caries and potential eating disorders. The study by Coffey et al. (84) further emphasized the impact of high sugar content in ONS products, which fosters the proliferation of cariogenic bacteria, exacerbating dental health issues. Meanwhile, research conducted by Theodorea et al. (85) in Indonesia identified an imbalance in *Veillonella* species among stunted children, correlating with poor oral hygiene and systemic health challenges. Lastly, a systematic review by Sadida et al. (86) found a strong association between stunted growth and oral health deterioration, marked by decreased salivary flow and altered saliva composition. These studies collectively underscore the intricate connection between nutritional status and oral microbiome health, highlighting the need for targeted interventions to improve both oral and overall health outcomes in malnourished children.

**Table 2:** Clinical studies of oral microbiome and malnourished children.

No.	Study	Population	Oral microbiome changes	Findings
1.	Osatogbe et al. (81)	Malnourished children, specifically protein-calorie malnutrition (PCM)	*Streptococcus* and *Enterococcus*↑	The findings reveal insights into the association between malnutrition, bacterial infections, and increased susceptibility to infectious diseases in malnourished children.
2.	Osatogbe et al. (82)	In-patient malnourished children at Sokoto Specialist Hospital in Nigeria	*Haemophilus influenzae*, *Staphylococcus aureus*, *Escherichia coli*, *Streptococcus pyogenes*, *Cronobacter condimenti*, *Photorhabdus luminescens*, *Klebsiella aeruginosa*, *Bacillus tequillensis*, *Yersinia molderath*, and *Bacillus megaterium*↑	Malnutrition disrupts the balance of the oral microbiome, leading to an overgrowth of pathogenic bacteria. These microorganisms contribute to conditions like periodontal disease, dental cavities, and systemic diseases such as respiratory infections and cardiovascular diseases.
3.	Fathallh et al. (83)	12-year-old children	*Streptococcus mutans*↑ *Streptococcus sobrinus*↑	More/less nutrient consumption (esp. carbohydrate/glucose) -> bacterial fermentation -> increase bacterial deposition on teeth -> adaptation of immune system through IgA to pathogen load -> salivary IgA increases -> biofilm formation -> fermentation induced the acidic oral condition -> enamel decalcification -> reduction of enamel microhardness -> dental caries -> chronic infection -> may cause disequilibrium metabolism and eating process (eating disorder)
4.	Coffey et al. (84)	Children with prescribed Oral Nutritional Supplements (ONS), focusing on their dental and oral implications	*Escherichia coli*, *Staphylococcus aureus*, and *Candida albicans*↑	High sugar content can promote cariogenic bacteria like *Streptococcus mutans*, which are associated with dental caries
5.	Theodorea et al. (85)	60 children (31 healthy children and 29 stunted children) aged 6–7 years from Nangapanda District, Ende, East Nusa Tenggara, Indonesia.	*Veillonella atypica*↑ *Veillonella denticariosi*↓ *Veillonella dispar*↓ *Veillonella parvula*↓ *Veillonella rogosae*↑ *Veillonella tobetsuensis*↑ *Veillonella infantium*↑	The imbalance in *Veillonella* species was associated with poor oral hygiene and nutritional status. Certain species like *V. denticariosi*, *V. infantium*, and *V. rogosae* were less abundant in stunted children, while *V. tobetsuensis* was more abundant in the stunted group. These imbalances potentially contribute to oral biofilm formation, reduced oral health, and systemic health challenges such as inflammation.
6.	Sadida et al. (86)	Systematic Review of Children aged up to 5 years old with a history of stunting	Systematic Review	There is a correlation between growth stunting and oral health in children. It was found that there was a decrease in salivary flow rate and the composition of saliva in children with growth stunting.

## 6 Implications of oral microbiome for stunting

### 6.1 Diagnostic approaches

The oral microbiome holds significant promise as a diagnostic tool for various health conditions, with recent studies emphasizing its intricate relationship with overall health. These findings suggest that oral microbial communities could serve as valuable biomarkers for conditions beyond the oral cavity (16). Accurate assessment of both the oral and gut microbiomes is particularly crucial in understanding their potential roles in childhood stunting. While extensive research has focused on the gut microbiome's impact on stunting, the role of the oral microbiome remains underexplored. However, emerging evidence points to its potential in providing biomarkers for early detection and intervention, underscoring the need for further investigation into this promising area (4, 87).

Traditional culture-based methods have limited utility in capturing the full diversity of the human microbiome. It involves growing microorganisms under specific conditions, such as optimal media, temperature, and pH, making it the gold standard for identifying living microbes and testing antibiotic susceptibility. Although it is economical and effective, but traditional culture is labor-intensive and unable to cultivate all organisms (39). In contrast, advanced molecular techniques, such as 16S rRNA gene sequencing and metagenomic analysis, have revolutionized the field by providing a more comprehensive and accurate representation of the microbial communities inhabiting the oral cavity and gastrointestinal tract. Among these, 16S rRNA amplicon sequencing stands out as the primary method for characterizing the oral bacteriome, utilizing a targeted gene approach. This technique offers an effective and straightforward alternative for conventional microbial culture methods, by analyzing the 16S rRNA gene, which encodes a ribosomal subunit present in most bacteria, with conserved regions and hypervariable regions. The hypervariable regions are unique to each bacterial species, enabling species identification and classification, while the conserved regions allow the design of universal primers that bind to shared sequences. Short-read sequencing of 200–400 base pairs can target specific hypervariable regions using primers aligned to conserved sequences. For instance, the V1–V3 region is commonly used in studies of the oral microbiome (88).

These techniques allow researchers to identify specific bacterial taxa, their relative abundances, and their functional capabilities, which can be correlated with clinical outcomes, including stunting. The core salivary microbiome in a healthy state predominantly includes bacterial phyla such as Actinobacteria, Bacteroidetes, Firmicutes, Proteobacteria, and Fusobacteria, with Streptococcus, Prevotella, and Neisseria being the most prevalent genera (89). Studies have also highlighted significant differences in microbial composition between healthy and malnourished or stunted children. For example, study of gut microbiota in Bangladesh revealed an increased prevalence of Proteobacteria and a decreased abundance of Bacteroidetes in malnourished children. Similarly, a study in Indonesia found that Prevotella 9 is positively correlated with children's linear growth, with a lower abundance of these bacteria in the stunted group compared to the non-stunted group (90).

Moreover, the identification of microbial biomarkers associated with childhood stunting may enable the development of non-invasive diagnostic tools for early detection and intervention. Biomarkers that correlated to stunting included plasma Immunoglobulin A anti-Lipopolysaccharide (IgA anti-LPS) and anti-FliC, zonulin (if over 12 months old), and intestinal fatty acid-binding protein (I-FABP), which indicate prior intestinal barrier disruption. Additionally, elevated levels of citrulline and tryptophan, along with low levels of serum amyloid A (SAA), were found, suggesting weakened immune defenses (91).

Another promising approach is the use of bio-indicators that reflect the functional effects of poor nutrition and environmental factors on human physical growth. These may include measures of intestinal damage, such as markers of gut permeability and systemic inflammation, which have been shown to precede and predict stunting in infants. Zonulin and intestinal fatty acid binding protein (IFABP/FABP-2) are markers for detecting intestinal permeability, while calprotectin and lipopolysaccharide (LPS) are associated with inflammation (92).

### 6.2 Interventions and treatments

Strategies to address childhood stunting must consider the complex interplay between the oral and gut microbiomes, alongside broader environmental and nutritional factors contributing to this global health challenge. Targeted interventions aimed at modulating these microbiomes, such as administration of probiotics, prebiotics, or antimicrobial therapies, show promise in promoting linear growth and improving nutrient absorption. For example, probiotics containing *Lactobacillus* and *Bifidobacterium* species have demonstrated potential in restoring microbial balance, enhancing gut barrier integrity, and reducing systemic inflammation. Similarly, prebiotics, including oligosaccharides, may support the growth of beneficial bacteria, indirectly influencing nutrient metabolism and growth outcomes (93). Table 3 shows the role of several probiotic, synbiotic, and prebiotic alternatives in stunting.

**Table 3:** Interventions and treatments of microbiomes on child.

No.	Interventions	Outcomes
1.	Probiotic (*Bifidobacterium. infantis*) or synbiotic (*B. infantis* EVC001 + Lacto-N-neotetraose)	Change in weight and length (94)
2.	RUTF and 4 g prebiotics	Change in weight (48)
3.	Synbiotic sachets contain 100 mg fructooligosaccharides and 150 million spore *Bacillus coagulans*	Change in wight, height, and head circumference (95)
4.	Synbiotic supplementation (109 CFU) 1 g daily	Change in weight, height, and BMI (96)
5.	Five drops (0.35 cc, approximately 109 microorganisms) of *L. rhamnosus* solution once a day	Change in BMI (97)
6.	Gummy *L. plantarum* Dad-13 (108–9 CFU/3g)	Change in weight and height (98)
7.	Prebiotics other than oligosaccharides such as PUFAs	Change in weight and height (99)
8.	Fermented milk 65 ml/day containing 108 CFU/ml of *Lactobacillus casei* for the 12-week intervention period	Have the gut dysbiosis (100)
9.	Lipid-based nutrient supplement enriched with prebiotics (LNSp)	Effects on metabolic activity (101)
10.	Daily administration of commercial U.S. donor–derived *B. infantis* strain (EVC001) with or without the HMO lacto-N-neotetraose for 28 days	Change in weight (102)
11.	Microbiota-directed complementary food prototype (MDCF-2) or a RUSF given twice daily for three months	Change in weight-for-length, weight-for-age, and length-for-age z scores and mid–upper-arm circumference (103)

CFU, colony forming unit; HMO, human milk oligosaccharides; RUSF, ready-to-use supplementary food; RUTF, ready-to-use therapeutic foods.

However, these approaches must be carefully considered, as disruptions to the microbial communities can have unintended consequences, such as dysbiosis or the proliferation of pathogenic bacteria, potentially exacerbating the problem (93). Moreover, these strategies should be integrated into holistic interventions that address underlying determinants of stunting, including food security, sanitation, and access to healthcare. Comprehensive research is required to refine these therapeutic strategies, ensuring their efficacy and safety while considering regional and individual variations in microbiome composition and environmental factors.

### 6.3 Prevention strategie

In addition to microbiome-based interventions, a holistic approach to addressing childhood stunting is essential. This includes improving maternal nutrition, promoting exclusive breastfeeding and appropriate complementary feeding practices, enhancing access to clean water, sanitation, and hygiene, and integrating these multifaceted strategies for a comprehensive solution. Improving maternal nutrition during pregnancy and lactation is crucial, as maternal undernutrition is a key driver of intrauterine growth retardation, which in turn contributes to stunting in early childhood (57). Maternal nutrition or diet plays a vital role in shaping the diversity of the infant's microbiome, with this effect not only occurring post-birth but also influencing in the intrauterine environment. This suggests that microbial diversity in the infant's gut is strongly influenced by maternal factors, including the mother's diet, which can modulate the colonization of beneficial bacteria and subsequently shape the infant's immune system and metabolic functions (76).

Maternal diet exerts a prolonged impact by influencing the diversity of the breastmilk microbiota, which can, in turn, affect the infant's nutritional status (77). Therefore, promoting exclusive breastfeeding and appropriate complementary feeding practices is crucial, as these practices help shape the development of the infant's microbiome and support optimal growth and development. It was estimated that around 1.6 million deaths and 105 million disability-adjusted life years (DALYs) were attributed to inadequate water, sanitation, and hygiene (WASH). By increasing access to adequate WASH, especially in low-middle-income countries, hopefully, the negative effects caused by infectious diseases in children, including stunting and other growth or developmental problems, can be significantly reduced. Integrating these multifaceted approaches, grounded in a robust understanding of the role of the oral and gut microbiomes, may hold the key to effectively preventing and addressing the global challenge of childhood stunting. These strategies hopefully can make a difference in the global stunting burden by promoting healthy oral and gut microbiomes for optimal nutritional absorption and preventing diseases that may hinder children's growth (68).

### 6.4 Implications for public health

Stunting is known as a global public health issue, affecting various stages of life, especially in the early stages of a child's life. Inhibition or obstacles to growth and development psychologically will affect the child's role and interaction in the social environment and response to stimulation as an adaptation to various changes, with the aim of increasing hope and quality of life (104). In physiological factors, these disorders cause various inhibitions or delays in the development of organelles or the individual as a whole, especially the cognitive system and susceptibility to various infections. Through these various vulnerabilities, parents (paternal-maternal) and children become important cycles for evaluation, followed by an environment that has various types of exposure. The preconception, gestation, and post-partum phases are important stages for determining promotive, preventive, and curative actions, especially related to nutrition and various risk factors, including comorbidities. Again, this factor essentially pays attention to the mother (maternal), but the father (paternal) contributes to these preparations. Appropriate interventions will provide a good level of life expectancy for children to be able to grow and develop, especially adaptation to various stimulations and changes that are assumed to be found differently in each individual (105).

In addition, microbiota in the digestive system, one of them is the oral cavity, affects enzyme mechanisms, nutrient absorption, and susceptibility to infection. In children, the risk of tooth decay increases based on food type and economic status. Consumption of high-calorie and sugar products raises the risk of tooth decay significantly, increasing the risk of stunting by 1.8 times. In addition, low-calorie, low-glucose, and high-fibre consumption raise saliva production that plays a close role in bad breath (halitosis), carbohydrate metabolism (amylase), and even antibacterial properties such as lysozymes, lactoferrin, and hydrogen peroxide. Several enzymes have been reported to be closely correlated with the risk of tooth decay, such as amylase. This disorder is one of the comorbidities of malnutrition, influenced by sociocultural factors and nutrition, as the etiology of toothache simultaneously causes a decrease in appetite and increases the risk of diabetes. Diabetes contributes to the risk of stunting, linked with fast-food consumption and high-calorie intake, which causes nutritional status impairment. Therefore, cases of nutritional disorders and oral microbiome play a role in various detrimental conditions that occur simultaneously or sequentially (106).

In conclusion, the emerging evidence on the role of the oral and gut microbiomes in childhood stunting underscores the need for a comprehensive, multidisciplinary approach to addressing this pressing global health issue. Advances in microbiome research, coupled with a deeper understanding of the complex interplay between nutrition, environment, and host physiology, can inform the development of innovative diagnostic tools and targeted interventions to promote optimal growth and development in children worldwide (69, 70).

### 6.5 Future directions

The field of oral and gut microbiome research in the context of childhood stunting is rapidly evolving, and there are several promising avenues for future exploration: Longitudinal studies that track the development of the oral and gut microbiomes from birth through early childhood, and their association with linear growth, can provide crucial insights into the timing and dynamics of microbial colonization and its impact on stunting. Such study could reveal the critical windows during which microbial composition may significantly influence linear growth and identify key microbial functions that serve as predictors or mediators of stunting. For instance, examining how early-life exposures—such as mode of delivery, maternal microbiome composition, breastfeeding practices, and dietary transition—affect microbial diversity and stability would provide foundation for early detection and targeted interventions.

Comparative studies across different geographic regions and cultural contexts can shed light on the role of environmental and dietary factors in shaping the microbiome and its relationship with stunting. Dietary variation, sanitary practices, and exposure to environmental pathogens can influence microbial composition and diversity that may influence child growth outcomes. For example, studies comparing rural and urban populations may identify how access to diverse food or clean water affects microbiome composition and functions. Such study can inform culturally and regionally appropriate intervention.

Interdisciplinary research integrating microbiology, nutrition, immunology, and environmental health can elucidate the complex, multifactorial pathways that contribute to stunting, informing the development of more effective, holistic interventions. For instance, understanding how disruptions of microbiome in the oral cavity influence systemic immune responses. This will provide insights into how dysbiosis contributes to both malnutrition and stunting. Such a holistic approach can help identify not only the link between microbial impacts on nutrient absorption, but also indirect relationships mediated through immune modulation and inflammation.

The prospective application of the oral microbiome in enhancing human health hinges on the rigorous validation of microbial biomarkers specific to various diseases. These biomarkers must be effectively integrated into diagnostic and preventive frameworks that are not only sensitive and specific but also deliver rapid results and are cost-effective for widespread use. When combined with advancements in human genomics, proteomics, transcriptomics, and metabolomics, the oral microbiome in children could become a pivotal element in the future of precision and personalized medicine.

The review paper has covered the key aspects of the relationship between the oral microbiome and childhood stunting, including the proposed mechanisms, diagnostic approaches, interventions, prevention strategies, and implications for public health. The paper has also highlighted important areas for future research to further advance our understanding of this critical global health challenge.

## 7 Conclusion

The oral microbiome and its interactions with the gut microbiome play a significant role in childhood stunting, a pervasive global health problem. Understanding the establishment and dynamics of the oral and gut microbiomes, as well as their functional impacts on growth and development, is crucial for developing effective diagnostic tools and targeted interventions to address this complex issue. The field of microbiome research has evolved rapidly over the past few decades and has become a topic of great scientific and public interest. Moreover, the oral microbiome can influence the gut microbiome through the gastrointestinal tract, a phenomenon known as the oral-cavity-driven gut microbiome, as the two are closely interconnected. Disruptions in the oral microbiome may have downstream effects on the gut microbiome, potentially leading to further impairment of nutrient absorption and immune function, both of which are key determinants of linear growth in children. Emphasizing the importance of oral microbiome, this study reviews the understanding of the oral microbiome's role in stunting that can direct novel diagnosis and treatment approaches.
